# Neuronal differentiation induces SNORD115 expression and is accompanied by post-transcriptional changes of serotonin receptor 2c mRNA

**DOI:** 10.1038/s41598-018-23293-7

**Published:** 2018-03-23

**Authors:** Tomaž Bratkovič, Miha Modic, Germán Camargo Ortega, Micha Drukker, Boris Rogelj

**Affiliations:** 1 University of Ljubljana, Faculty of Pharmacy, Department of Pharmaceutical Biology, Aškerčeva 7, 1000 Ljubljana, Slovenia; 2 Institute of Stem Cell Research and the Induced Pluripotent Stem Cell Core Facility, Helmholtz Center Munich, 85764 Neuherberg, Germany; 3 Jozef Stefan Institute, Department of Biotechnology, Jamova 39, 1000 Ljubljana, Slovenia; 4 Biomedical Research Institute BRIS, Puhova 10, 1000 Ljubljana, Slovenia; 5 University of Ljubljana, Faculty of Chemistry and Chemical Technology, Večna pot 113, 1000 Ljubljana, Slovenia; 6 Physiological Genomics, Biomedical Center, Ludwig-Maximilian University Munich, Munich, Germany

## Abstract

The serotonin neurotransmitter system is widespread in the brain and implicated in modulation of neuronal responses to other neurotransmitters. Among 14 serotonin receptor subtypes, 5-HT2cR plays a pivotal role in controlling neuronal network excitability. Serotonergic activity conveyed through receptor 5-HT2cR is regulated post-transcriptionally via two mechanisms, alternative splicing and A-to-I RNA editing. Brain-specific small nucleolar RNA SNORD115 harbours a phylogenetically conserved 18-nucleotide antisense element with perfect complementarity to the region of *5ht2c* primary transcript that undergoes post-transcriptional changes. Previous *5ht2c* minigene studies have implicated SNORD115 in fine-tuning of both post-transcriptional events. We monitored post-transcriptional changes of endogenous *5ht2c* transcripts during neuronal differentiation. Both SNORD115 and *5ht2c* were upregulated upon neuronal commitment. We detected increased *5ht2c* alternative exon Vb inclusion already at the stage of neuronal progenitors, and more extensive A-to-I editing of non-targeted sites A and B compared to adjacent adenosines at sites E, C and D throughout differentiation. As the extent of editing is known to positively correlate with exon Vb usage while it reduces receptor functionality, our data support the model where SNORD115 directly promotes alternative exon inclusion without the requirement for conversion of key adenosines to inosines, thereby favouring production of full-length receptor isoforms with higher potency.

## Introduction

**Figure 1:**
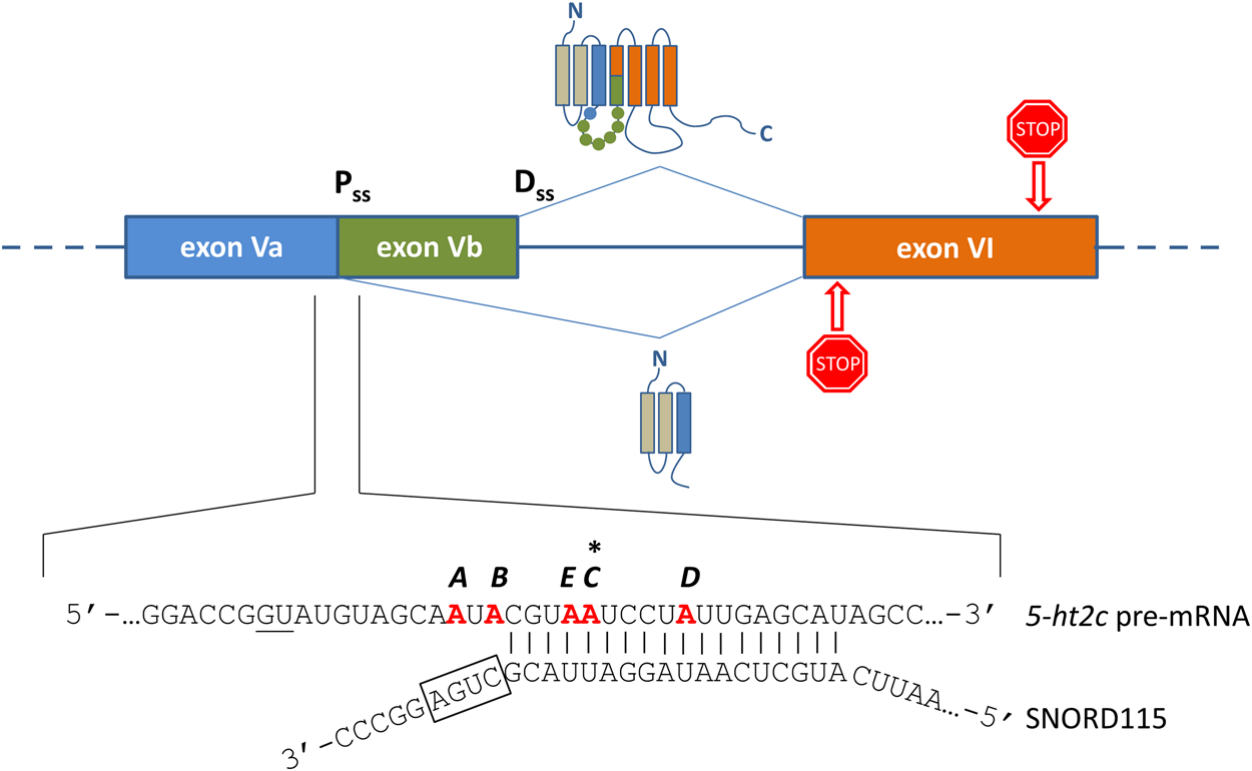
Depiction of alternative splicing and A-to-I editing positions (denoted A to E) within the *5ht2c* transcript. Stop signs symbolize stop codons. GU dinucleotide of the alternative 5′ donor splice site is underlined. RNA duplex is formed between an 18-nucleotide *5ht2c* segment and SNORD115 antisense region. Characteristic nucleotide motif in SNORD115 (the D-box) is boxed and adenosine on *5ht2c* transcript located 5 nucleotides upstream, which is presumed to be targeted for 2′-O-methylation (coinciding with the editing position C), is denoted by an asterisk. Pss, proximal splice site; Dss, distal splice site.

Serotonin receptor 2 C (5-HT2cR) is a member of the 7-transmembrane-spanning receptors that convey signals through coupling with intracellular guanine nucleotide-binding proteins (G proteins). It is expressed almost exclusively in the central nervous system, where its activity is associated with control over neuronal network excitability. 5-HT2cR was implicated in regulation of a variety of physiological functions such as mood, satiety and reproduction, and aberrant 5-HT2cR signalling might underlie anxiety, depression, schizophrenia, and obesity^1^. Regulation of 5-HT2cR signalling is highly complex and it is recognized that receptor activity is controlled at several regulatory layers^2^. Adenosine deaminases acting on RNA (ADARs) convert specific adenosines in the *5ht2c* primary transcript to inosines (the phenomenon is known as A-to-I editing). As inosine is recognized by translational machinery as guanine, this diversifies the genetic code post-transcriptionally, resulting in amino acid substitutions with regard to the genetically encoded receptor^3^. There are five closely spaced adenosines (denoted A-E) with editing potential contained within the alternative exon Vb (Fig. 1). ADAR enzymes act on the double-stranded RNA structure resulting from intron V folding back on exon Vb; ADAR1 preferentially edits positions A and B, whereas position D is selectively edited by ADAR2; positions E and C are deaminated by both enzymes^4^. Exon Vb encodes the second intracellular loop through which the receptor couples to G proteins^3^. Functional characterization of 5-HT2cR isoforms produced as a consequence of A-to-I editing revealed diminished receptor constitutive activity^5–8^, potency^3,8,9^, and agonist affinity^10^ as compared to the unedited receptor. Furthermore, editing seems to be regulated in a serotonin-dependent manner as mice treated with a serotonin re-uptake inhibitor display perturbed A-to-I editing resulting in expression of receptor isoforms with higher serotonin sensitivity^11^. Finally, *5ht2c* primary transcript is subjected to alternative splicing^12^, whereby skipping of the alternative exon Vb leads to translational shift resulting in an early stop codon, terminating translation after the third transmembrane domain (Fig. 1). Both mRNA isoforms were detected in various regions of mouse and human brain at different ratios^8,12^ and thus appear to be stable. An *in vitro* study on cells transfected with cDNA of both splice receptor isoforms revealed that the truncated receptor localizes in the endoplasmic reticulum where it sequesters the full-length isoform, preventing it to reach plasma membrane^13^. Recently, Zhang *et al*. confirmed the existence of truncated receptor isoform in mouse brain^14^. It has been shown that editing has a deciding role in splice site selection^13,15^.

Alternative exon Vb contains a phylogenetically conserved 18-nucleotide sequence with perfect complementarity to the brain-specific C/D-box small nucleolar RNA (snoRNA) SNORD115^16^ (Fig. 1), suggesting its involvement in the fine tuning of serotonergic signal transduction. snoRNAs are a group of short non-coding RNAs principally involved in post-transcriptional modification of ribosomal and small nuclear RNAs^17^. As a part of ribonucleoprotein complexes, C/D-box snoRNAs specify individual nucleotides of substrate RNAs for 2′-O-ribose methylation by partner enzyme fibrillarin through Watson-Crick base pairing. In humans, SNORD115 genes constitute an intronic cluster within the paternally-imprinted locus 15q11-q13 (syntenic region in mice is found on chromosome 7) that harbours, in addition to protein-coding genes, several other conserved orphan snoRNAs, including SNORD116. Orphan snoRNAs lack any obvious base-pairing with the canonical target RNAs, and their biological role remains elusive. Deletions in the 15q11-q13 locus^18–21^ were identified as the genetic cause of Prader-Willi syndrome (PWS), a rare neurological disorder, characterized by developmental, behavioural, and psychiatric problems. Subjects with PWS present some features which can be explained by dysregulation of serotonergic system, such as obsessive-compulsive behaviour, and selective serotonin re-uptake inhibitors are sometimes prescribed to ameliorate the symptoms^22,23^. Although recently a microdeletion encompassing the entire SNORD116 gene cluster was determined as the minimal requirement underlying PWS phenotype^21^, lack of SNORD115 expression likely contributes to the disorder.

The most compelling evidence implicating SNORD115 in modification of *5ht2c* pre-mRNA comes from studies on cell models. However, whereas Vitali *et al*.^4^ showed that SNORD115-guided ribose methylation inhibits A-to-I editing selectively at position C, Kishore and Stamm^24^ demonstrated that SNORD115 affects alternative splicing in a methylation-independent manner, plausibly by transiently associating with a silencer element at the proximal splice site to promote exon Vb inclusion. These studies were performed on neuroblasts and/or fibroblasts transiently transfected with engineered *5ht2c* minigenes that were either artificially targeted to nucleolus^4^, the site of SNORD115 accumulation, or had their distal splice site (Fig. 1) mutated to mammalian consensus to activate splicing in immortalized cell line^24^. The difference in technical setup might explain contrasting conclusions on the biological role of SNORD115. It would thus be highly informative to examine the post-transcriptional changes of the wild-type *5ht2c* gene expressed from the native promoter.

However, ectopic expression of full length *5ht2c* is difficult due to gene spanning over 326 kb. On the other hand, functional studies of snoRNAs are hampered by the fact that they are highly structured and shielded by partner proteins within the nucleolar ribonucleoprotein complexes, rendering gene knock-down approaches difficult to implement^25,26^. Thus, reports of successful snoRNA knock-down experiments are scarce^25,27,28^. More importantly, SNORD115 knock-down is complicated by redundancy as there are numerous homologous copies of the gene.

By analysing recent RNA-seq data on whole transcriptome profiling of murine embryonic carcinoma cell line P19^29^, we observed that both SNORD115 gene cluster and *5ht2c* are strongly upregulated following retinoic acid-induced neuronal differentiation. To get insight into dynamic post-transcriptional processes affecting *5ht2c* transcript during neuroectodermal differentiation in conjunction with increased expression of SNORD115, we monitored its splicing and A-to-I editing profiles throughout transition of pluripotent P19 cells to neurons.

## Results

### Choice of cell line and induction of neuronal differentiation

**Figure 2:**
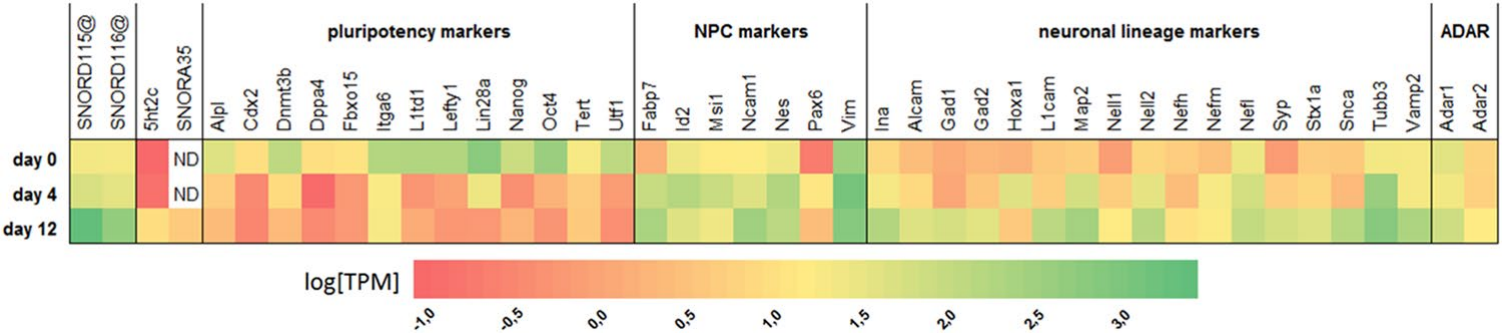
Heat map of expression levels (log-transformed transcript per kilobase million (TPM) values) for selected genes during the course of retinoic acid-induced neural differentiation of P19 cells. Figure was generated based on RNA-seq data from Rybak-Wolf *et al*.^29^. NPC, neural progenitor cell; ND, not detected.

RNA-seq analysis (Fig. 2) built on available dataset^29^ showed that neurons produced from P19 cell line express SNORD115 and *5ht2c* genes at levels up to 2 orders of magnitude higher than the neuronal precursors with neuronal *5ht2c* transcripts being 200-fold underrepresented compared to combined SNORD115 cluster members (it should be noted, however, that the seemingly low *5ht2c* expression might in fact be due to extensive A-to-I editing which prevents the correct annotation of edited reads). Differentiating P19 cells were thus chosen as a model system to monitor *5ht2c* post-transcriptional modifications in correlation with SNORD115 expression changes.

**Figure 3:**
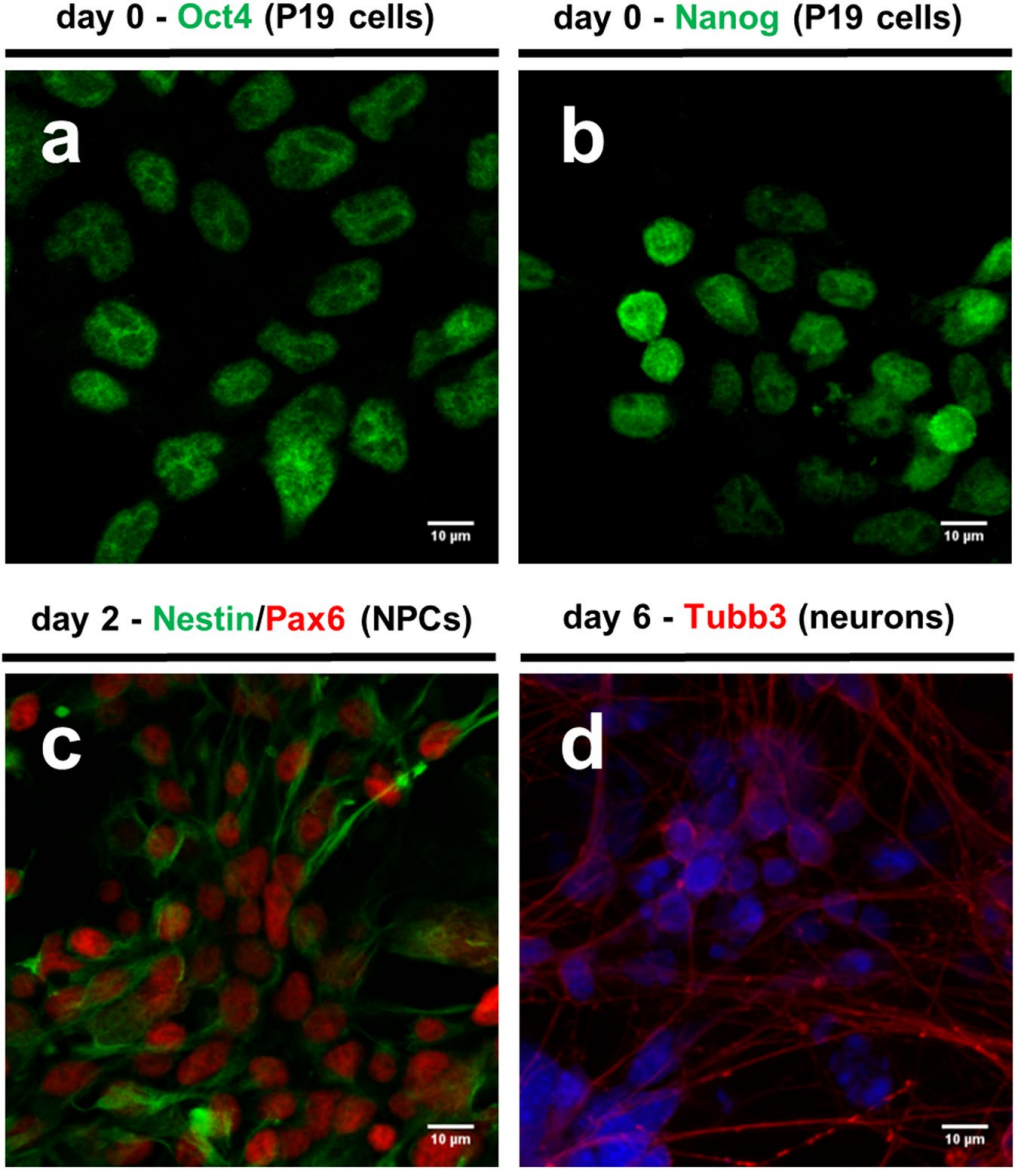
*In vitro* differentiation of P19 cells mimics early processes in mammalian neuroectodermal development *in vivo*. (**a**) and (**b**) Confocal images of P19 cells stained with anti-Oct4 and anti-NANOG antibody (FITC-green), respectively. (**c**) Confocal images of P19-derived neural progenitor cells (NPCs) on the second day of neuronal induction upon plating on adhesive tissue culture dishes stained with anti-Pax6 (FITC-green) and anti-nestin (FITC-Texas). (**d**) Neuronal precursors become post-mitotic and undergo end-terminal differentiation, observed by confocal microscopy of neurons stained with anti-Tubb3 on day 6 of neuronal differentiation upon plating on adhesive tissue culture dishes. Cell nuclei were counterstained with DAPI. Images of immuno-staining and DAPI staining were overlaid. Scale bar represents 10 micrometers.

Neuronal differentiation of pluripotent cell line P19 was triggered by treating floating cell aggregates with RA and finally plating trypsinized cells on poly-D-lysine plates. Cells at progressing stages of differentiation were stained with antibodies against a set of markers, confirming differentiation status (Fig. 3). Neural progenitors (Pax6 + /Nestin + ) were enriched on the second day of neuronal induction (i.e. upon plating on adhesive tissue culture dishes), and neurons (Tubb3 + ) appeared from day 4 of differentiation onwards. Throughout differentiation a homogenous population of cells was observed, in line with a previous report on neuronal induction of murine embryonic stem cells using an analogous protocol^30^, and no selective trypsinization was needed to enrich for neuronal cells.

### qPCR analyses of gene expression changes during neuronal differentiation

**Figure 4:**
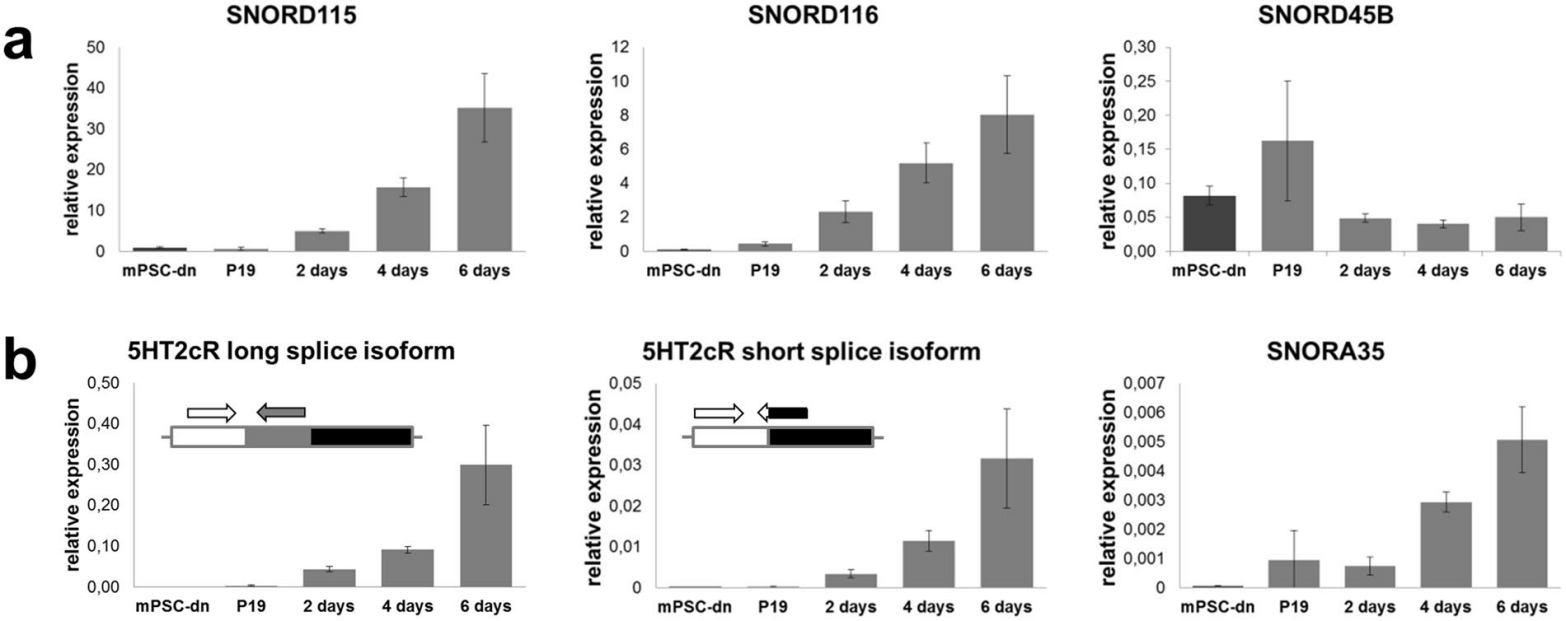
snoRNAs and *5ht2c* mRNAs levels are gradually upregulated during neuronal commitment. (**a**) RA-induced neuronal differentiation unlocks expression of PWS locus snoRNAs SNORD115 and SNORD116, but does not affect expression of canonical snoRNA SNORD45B. (**b**) Neuronal differentiation induces expression of both serotonin receptor 2c splice variants as well as SNORA35, snoRNA harboured in the second intron of *5ht2c* gene. Primer design is depicted for *5ht2c* splice variants. Averaged data of three biological repeats per time point ± standard deviation are shown. mPSC-dn, murine pluripotent stem cell-derived neurons; X days, time after induction of neuronal differentiation.

In mice, SNORD115 and SNORD116 are encoded in large intronic clusters of multiple highly homologous copies. We semi-quantitatively analysed SNORD115/116 expression in murine pluripotent stem cell (mPSC)-derived neurons and retinoic acid (RA)-induced P19-neurons by qPCR. Whereas SNORD115 and SNORD116 were not detected in mPSC-derived neurons, they were strongly induced by RA and their expression levels rose with neuronal differentiation stage (Fig. 4a). In comparison, the level of SNORD45B, a canonical snoRNA predicted to guide the 2′-O-ribose methylation at U172 of 18 S rRNA^31^, was unaffected by RA treatment (Fig. 4a). We next looked at the expression of *5ht2c* and found similar profiles to PWS locus snoRNAs; *5ht2c* mRNA was absent in mPSC-derived neurons, very poorly expressed in P19 cell line, but induced by RA (Fig. 4b). Primers for *5ht2c* long splice variant quantification were positioned so as to amplify the cDNA isoform regardless of editing. Both splice variants were readily detected and had qualitatively similar expression profiles. Finally, we determined expression of SNORA35 (Fig. 4b), a brain-specific snoRNA harboured in the second intron of *5ht2c* gene. Some pre-mRNAs have been shown to transiently localize to the nucleolus^32^. The presence of intronic snoRNA in the *5ht2c* gene could suggest that the primary transcript is trafficked through the nucleolus where it may encounter SNORD115. Qualitatively, SNORA35 displayed similar profiles to those of *5ht2c* and PWS snoRNAs but was expressed at significantly lower degree in all cells analysed (see also Fig. 2). The qPCR data are in agreement with gene expression profiles previously determined with RNA-seq^29^.

### The proportion of *5ht2c* long splice isoform increases upon neuronal commitment

**Figure 5:**
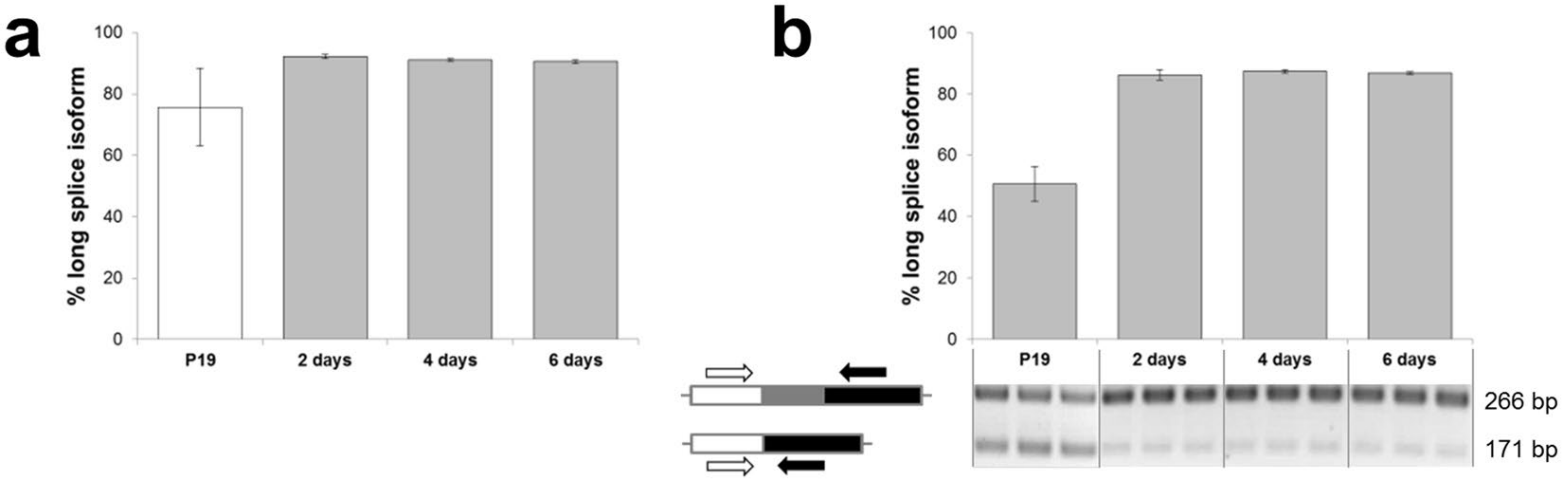
Analysis of splicing for the *5ht2c* transcript in P19 cells and during RA-induced neuronal differentiation. (**a**) Percentage of long splice isoforms as inferred from the qPCR expression data. See Fig. 4b for depiction of primer pairs used to detect the two isoforms. White column denotes low reliability of data due to extremely low *5ht2c* expression. (**b**) Percentage of long splice isoforms as deduced by densitometric quantification of RT-PCR amplicons corresponding to the long and short splice isoforms. Averaged data of three biological repeats per time point ± standard deviation are shown.

Only the long *5ht2c* splice isoform is translated to receptor capable of signal transmission, whereas the short splice isoform gives rise to truncated receptor sequestering the full length receptor intracellularly^13,14^. To examine whether *5ht2c* splicing changes are coupled with neuronal differentiation, we assessed the alternative exon Vb usage in P19 cells during the time course of neuronal differentiation. At pluripotency stage the P19 cells transcribe extremely low levels of *5ht2c* (Fig. 4b), making quantification of splicing challenging. Only upon induction of differentiation, when *5ht2c* expression is significantly increased, can quantitative splicing changes be determined. As deduced from qPCR data, the ratio of long to short isoform remained constant from neural progenitors to neurons (92.2 ± 0.8, 91.1 ± 0.5, 90.5 ± 0.6 percent, respectively, in favour of the long isoform; Fig. 5a). We re-analysed the splicing profile by a complementary technique, RT-PCR and densitometric quantification of amplicons (Fig. 5b). Here, the ratio of amplicons of cognate isoforms should reflect their initial proportion irrespective of the input amount. Nevertheless, we used 35-fold more cDNA per PCR reaction to ensure that the rare transcripts were not overlooked. With this approach we confirmed that the proportion of long splice isoform was highly comparable among later time points (i.e., 86.2 ± 1.6, 87.3 ± 0.5, 86.7 ± 0.4 percent, respectively). In contrast, we observed a significantly smaller proportion of long splice isoform in pluripotent P19 cells (50.9 ± 5.9 percent; p < 0.0001, one-way ANOVA with Bonferroni *post hoc* test). These data indicate that the splicing profile of *5ht2c* shifts towards full length isoform once the cells commit to neuronal differentiation.

### The extent of *5ht2c* A-to-I editing is low at sites overlapping with SNORD115 binding region

**Figure 6:**
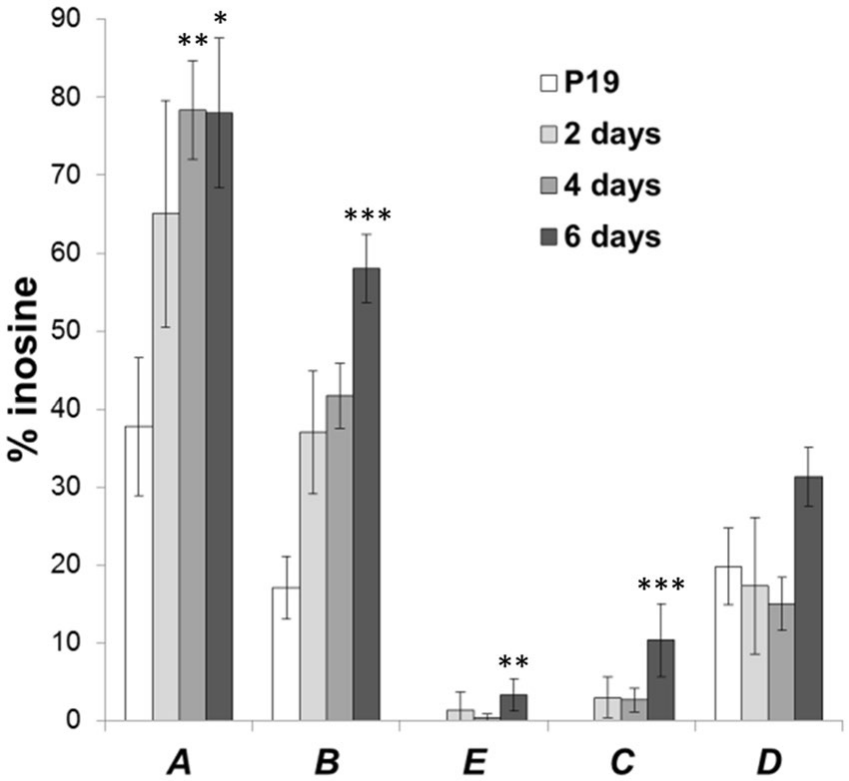
Determination of A-to-I editing levels by direct PCR sequencing. Averaged data ± standard deviation are shown. Statistically significant changes in the extent of editing (relative to undifferentiated P19 cells) are denoted by asterisks (Kruskal-Wallis one-way analysis of variance with Dunn’s post *hoc test*; *P_adjusted_ < 0.1; **P_adjusted_ < 0.05; ***P_adjusted_ < 0.01).

Having observed changes in alternative splicing of *5ht2c* transcript, we next quantified A-to-I editing levels at each of the 5 editing sites during RA-induced neuronal differentiation (Fig. 6 and Supplementary Figure S1). Although direct amplicon sequencing does not support analysis of individual mRNA variants, it is convenient for assessing the overall editing levels at individual nucleotide positions^33^. During preliminary qPCR gene expression profiling we determined that in neurons, expression levels of adenosine deaminases ADAR1 and ADAR2 are approximately 2- and 3-fold higher, respectively, compared to the pluripotency stage (Supplementary Figure 2); we stress, however, that the primers for quantification of ADAR transcripts were designed so as not to discriminate among numerous ADAR splice isoforms and thus gene expression levels do not necessarily correlate with enzymatic activity.

We noted that at positions A and B, the only two sites not overlapping with the SNORD115 antisense element (Fig. 1), editing levels were highest already in P19 cells and further increased throughout neuronal differentiation to reach 78 ± 10% and 58 ± 4%, respectively. At position D editing was less pronounced, amounting to 31 ± 4%, whereas adenosines at sites E and C were deaminated only to a minor extent (i.e., 3 ± 2% and 10 ± 5%, respectively, in neurons). The differentiation-related increase in editing levels was observed for all positions with the exception of D (Fig. 6).

We estimated the accuracy of A-to-I editing determination. Plasmid-harboured single *5ht2c* fragment clones differing in editing at 3 positions were mixed in different ratios and sequenced. Guanosine peak hights relative to the sum of adenosine and guanosine peak heights were plotted against the defined G to A ratio (Supplementary Figure 3). For G to A ratios in the range of 20:80 to 80:20, the curve is linear. In this range, G content is overestimated by roughly 10 percentage points. At lower and higher G to A ratios, the shape of the calibration curve turns sigmoidal, allowing somewhat less accurate determination of editing extent; an observation, that is consistent with findings of Wahlstedt *et al*.^34^. Thus, editing, as analysed here, is likely slightly overestimated at positions A and B, and underestimated at sites E and C. Nevertheless, less extensive editing is evident for positions E, C and D relative to that at sites A and B.

## Discussion

The studies by Vitali *et al*.^4^ and Kishore and Stamm^24^ addressed the potential role of SNORD115 in regulation of post-transcriptional events of the *5ht2c* transcript in cells cotransfected with engineered *5ht2c* minigenes and SNORD115. Of note, in these experiments the minigenes were either artificially targeted to nucleolus (where SNORD115 accumulates) by means of RNA polymerase I promoter, or the experimenters relied on mutated distal splice site to achieve efficient splicing of minigenes in immortalized cell line. As manipulating *5ht2c* gene might have skewed the interpretation of observed effect (indeed, the studies attributed distinct roles to SNORD115 with regards to post-transcriptional processing of *5ht2c*), we monitored alternative splicing and A-to-I editing profiles of endogenously expressed serotonin receptor 2c transcript throughout retinoic acid-induced neuronal differentiation of murine embryonic carcinoma cell line P19 that concomitantly expresses SNORD115.

Over the course of neuronal differentiation the expression of both SNORD115 and *5ht2c* was strongly induced. We observed an increase in *5ht2c* alternative exon Vb usage upon neuronal commitment (Fig. 5). The levels of *ADAR* genes’ expression rose steadily with differentiation stage (Supplementary Figure 2), which is consistent with markedly increasing adenosine deamination at most editing sites (Fig. 6). Notably, positions that overlap with SNORD115 antisense element (i.e., C through E) were poorly edited. Tohda *et al*.^33^ who analysed editing of *5ht2c* mRNA in rat brain cortex during pre- and postnatal development, made observations similar to ours and speculated that factors other than ADAR enzymes must affect differential susceptibility of individual adenosines to deamination. One explanation for the observed phenomenon is that SNORD115-containing snoRNPs mask the distal editing sites, thereby competing with ADAR activity.

Site C adenosine is the nucleotide that was predicted to be targeted for 2′-O-ribose methylation by SNORD115-associated methyltransferase fibrillarin^16^ (Fig. 1). In *in vitro* experiments on synthetic substrates^35^ it has been demonstrated that deamination of adenosines with 2′-O-methylated ribose is greatly reduced. This does not, however, account for the low extent of editing observed at positions E and D. Instead, our data favour a model proposed by Kishore and Stamm^24^ in which SNORD115 directly promotes formation of *5ht2c* long splice isoform by masking the silencer of splicing element overlaping with the distal editing sites. In line with that, SNORD115 has been found to associate with non-canonical partner proteins^36,37^, further questioning its role in 2′-O-ribose methylation.

Flomen *et al*.^15^ performed a detailed analysis of the effect of adenosine deamination on splice site selection of *5ht2c*. By transfecting *5ht2c* minigenes spanning from exon Va to exon VI with combinations of adenosines at editing sites substituted for guanosines (simulating inosines) and quantifying exon Vb usage, they have shown that editing at positions A and B alone is insufficient to promote exon Vb retention. However, when editing at sites E, C, and/or D was simulated in addition to mutation of the proximal sites, the splicing equilibrium shifted markedly towards the long isoform. The shift can likely be attributed to alteration of the binding sites for trans-acting splice factors. As editing at positions C and E most strongly negatively influences 5-HT2cR receptor signalling^3,7,8^, SNORD115 seems to favour the generation of highly functional receptor isoforms by promoting exon Vb retention without the need for extensive editing^38^. Indeed, increased *5ht2c* mRNA editing was observed in post-mortem hippocampal regions of individuals suffering from PWS (lacking SNORD115 expression) but the splicing profile was indistinguishable from that of healthy controls^24^. On the other hand, a study performed on frontal cortices^39^ did not reveal consistent differences in the extent of *5ht2c* mRNA editing in PWS patients with confirmed SNORD115 deletion as compared to healthy controls. This discrepancy might be explained by the fact that editing was studied in different brain regions. Indeed, A-to-I editing is known to vary considerably across brain anatomy^8^.

Studies on transgenic animals corroborate the complex interplay between *5ht2c* splicing and A-to-I editing and support the role of SNORD115 in fine-tuning of both post-transcriptional events. In mice expressing solely the fully edited receptor isoform, the *5ht2c* short splice isoform was produced to a significantly lesser extent^13^, leading to diminished sequestration of the full length receptor in endoplasmic reticulum. These mice thus expressed more 5-HT2cR than the wild type animals, and even though the fully edited isoform has compromised signalling activity, displayed anxiety-like behaviour when treated with a preferential 5-HT2cR agonist. Interestingly, an earlier study^40^ on mice mutants expressing the fully edited receptor isoform found no differences in the *5ht2c* splicing profile compared to the wild-type mice. It did, however, confirm increased 5-HT2cR density in all analysed brain areas as judged by radioactively labelled receptor agonist binding. This observation is even more meaningful if one considers lower ligand affinity towards edited isoforms^10^. In transgenic mice with inactivated PWS locus (lacking SNORD115 expression) initially only increased editing but no changes in splicing profile of *5ht2c* pre-mRNA were found^41^, but a later more neuroanatomically refined analysis revealed a higher proportion of short splice isoform in the hypothalamus^42^.

Cell lines endogenously expressing *5ht2c* and SNORD115 have not been described. As RA-induced neuronal differentiation of P19 cells seems to faithfully recapitulate the processes that *5ht2c* transcript is subjected to in conjunction with SNORD115 upregulation, and since neuronal induction is fast and efficient, P19 cell line represents an interesting model system for studying PWS snoRNA functions. Given the role of 5-HT2cR in regulation of neuronal network excitability^1^, one can imagine that fine-tuning its activity should be of crucial importance for the developing neural system. In this respect, SNORD115 as a factor that both replaces and selectively counteracts ADAR activity at *5ht2c* transcript would be essential for normal neurogenesis.

Although not being sufficient for the onset of the disorder, SNORD115 cluster deletion is likely to augment the phenotypic characteristics of PWS through loss of the post-transcriptional fine-tuning mechanism of *5ht2c* transcript. Three studies strongly support this hypothesis. Mice lacking expression of PWS locus genes exhibited alterations in specific 5-HT2cR-related behaviours along with increased RNA-editing of the *5ht2c* pre-mRNA^41^. The same genetic mouse model of PWS was later shown to be unresponsive to anorexic doses of preferential 5-HT2cR agonist which was attributed to increased proportion of *5ht2c* short splice isoform in hypothalamic pro-opiomelanocortin neurons^42^. Furthermore, transgenic mice expressing solely fully edited 5-HT2cR isoform presented certain phenotypic traits of PWS, including failure to thrive, neonatal muscular hypotonia and reduced food consumption with subsequent (post-weaning) hyperphagia^43^. Etiology of PWS is complex and cannot be explained solely by the lack of SNORD115 expression, especially in light of recent evidence of SNORD116 deletion being responsible for sleep disorders^44^, growth retardation^45^, and body weight regulation^46,47^ in mice PWS models and/or PWS patients. Yet, there is mounting evidence to suggest an adjunctive role of SNORD115 in this genetic disorder. The exact role of PWS locus orphan snoRNAs remains to be determined. New and unexpected roles have been ascribed to several canonical and orphan snoRNAs in recent years (^27,48–52^ and reviewed in^53^), straitening our convictions that this class of non-coding RNAs is more than a simple regulator of rRNA decoration.

## Materials and Methods

### Global profiling of gene expression

Annotated RNA-seq data of P19 cell line undergoing neuronal differentiation (GEO accession numbers: GSM1665895, GSM1665896, GSM1665897^29^) with calculated TPM values were acquired from Rajewsky lab (Max Delbrück Center for Molecular Medicine, Berlin, Germany). A heat map (Fig. 2) was generated in Excel for SNORD115 and SNORD116 clusters, *5ht2c*, SNORA35, *ADAR1*, *ADAR2*, and sets of pluripotency, neural progenitor cell, and neuronal lineage markers for graphic presentation of expression changes during differentiation.

### Retinoic acid-induced neuronal differentiation of P19 cells

All experiments were performed on 3 biological replicates.

Pluripotent P19 cells (ATCC CRL1825) were cultured in growth medium (alphaMEM (Life Technologies), 10% fetal calf serum, 100 U/mL Penicillin and 100 µg/mL Streptomycin) at 37 °C and 5% CO2 and passaged every third day. Neural differentiation was induced by growing 3 × 10^6^ cells for four days on neural induction medium (alphaMEM, 5% FCS, 100 U/ml penicillin, 100 µg/mL streptomycin, 5 µM all-trans retinoic acid (RA)) on 100 mm nonadhesive bacterial petri dishes to promote cell aggregation. Afterwards, cell aggregates were collected, mechanically and enzymatically (0.05% trypsin) dissociated and plated on poly-D-lysine coated plates or coverslips and grown in neuronal induction medium (DMEM/F12 Glutamax (Life Technologies), 2% B27, 8 mM HEPES, 100 U/mL penicillin, 100 µg/mL streptomycin, 5 µM RA) for 6 days. We added 10 µM cytosine β-D-arabinofuranoside to the medium on the second day of neuronal differentiation in order to inhibit growth of dividing progenitors which would otherwise generate glia-like cells.

### Neural conversion of murine pluripotent stem cells

mPSC were differentiated from culture grown in monolayer without the step involving the formation of embryoid bodies or treatment by RA. First, mPSC were trypsinized, resuspended, quantified in a Neubauer counting chamber and transferred to uncoated 6-well cell culture plates. Following a 50-min-incubation period, supernatants were transferred to wells coated with poly-ornithine and laminin, maintaining cell densities at 2 × 10^4^ cells/cm2. Prior to initial dissociation step, 12 ng/mL bFGF was added to ESC culture medium (83% KnockOut DMEM, 15% KnockOut Serum Replacement (KSR), 0.1 mM non-essential amino acids, 1% Glutamax; Life Technologies) and incubated for 6 h in absence of LIF. Next, cells were dissociated and incubated in ROCK-Inhibitor (Y-27632; StemCell Technologies) in 10% KSR-medium (79% KnockOut DMEM, 20% KSR, 0.1 mM non-essential amino acids, 2 mM glutamine, 1 × 2-mercaptoethanol (Millipore)), supplemented with LDN193189 (LDN; 100 nM) and SB431542 (SB; 10 µM). At day 1 of differentiation, medium was changed to 5% KSR and 0.5% N2-supplement with PD0325901 (PD; 1 µM) on top of before listed inhibitors. Neural conversion was induced using the following protocol. On day 4, medium was changed to N2 based medium (DMEM/F-12 Glutamax, 1 × N2-supplement, 200 µM ascorbic acid) supplemented with 100 ng/ml FGF8, 100 ng/ml WNT1, 100 nM LDN and 10 µM SB. At day 6, medium was replaced with N2/B27 medium (50% DMEM/F-12 Glutamax, 50% Neurobasal Medium, 0.5 × N2-supplement, 0.5 × B27-supplement, 200 µM ascorbic acid) supplemented with 100 ng/ml FGF8, 100 ng/ml WNT1 and 100 nM LDN. FGF8 was omitted on day 8 from neuronal induction culture, and from this day onwards cells were differentiated in B27 medium (Neurobasal Medium, 1 × B27-supplement, 2 mM glutamine, 200 µM ascorbic acid) with medium being changed every two days using the following supplements (from day 8 to day 9: 100 ng/ml WNT1 and 100 nM LDN; from day 10 to day 11: 100 ng/ml WNT1). From day 11 of differentiation onwards, only BDNF (20 ng/ml) was added to B27 medium and differentiation was stopped at day 20.

### Immunocytochemistry

Cell markers were stained with antibodies given in Table 1. Imaging was performed with an Olympus FV1000 cLSM/MPE system, composed of a BX- 61WI upright microscope (Olympus), a laser diode 559 nm, a multi-line Argon laser (458 nm, 488 nm, Showa Optronics Co.) and internal photomultiplier tube detectors. Image analysis was performed using the FW10-ASW 4.0 software (Olympus).

**Table 1:** Antibodies used for immunocytochemistry.

Antigen	Host/IgG	Dilution	Clone	Company	Cat. No.
Oct3/4	mouse IgG2b	1:500	C-10	SantaCruz	sc-5279
Nanog	rabbit	1:500	polyclonal	CellSignaling	D73G4
Pax6	rabbit	1:500	polyclonal	Millipore	AB2237
Nestin	mouse IgG1	1:500	rat-401	Millipore	MAB353
Tubb3	mouse IgG2b	1:2000	SDL.3D10	SigmaAldrich	T8660

### qPCR analysis of gene expression

RNA was isolated from P19 cells at different time points of neuronal differentiation (0, 2, 4, and 6 days after plating cells on poly-D-lysine-coated plates) using the miRNeasy kit (Qiagen, Venlo, Netherlands). For mPSC-derived neurons, RNA was isolated from fully differentiated culture only. 1 µg of total RNA was reverse transcribed with random primers in 10 µL reactions using the SuperScript VILO cDNA Synthesis Kit (Thermo Fisher Scientific, Waltham, MA, USA) following the manufacturer recommendations. Gene expression was profiled with qPCR using Maxima SYBR Green qPCR Master Mix (Thermo Fisher Scientific) on LightCycler 480 (Roche, Basel, Switzerland). Primers (Table 2) were either acquired from literature or designed for the purpose of this study based on gene sequences accessed from Ensembl Genomic Browser. 0.63 ng cDNA was used per 10 µL reaction. Expression levels were normalized to *Actb* and *HPRT* mRNA, which were indicated as most stably expressed among 5 tested potential reference genes (*Actb*, *HPRT*, *SNORD45B*, *RPL27* and *Tubb3*) by NormFinder^54^. Reactions were analysed by agarose gel electrophoresis to confirm the presence of a single amplicon and reaction specificities were backed up by melting curve analysis. No reverse transcription controls indicated negligible extent of genomic DNA contamination of RNA isolates (Supplementary Figure 4).

**Table 2:** Primer pairs used for qPCR analysis of gene expression. All primers were used at 300 nM, except for 5HT2c_long/short pairs, which were used at 200 nM.

Gene	Primer pairs	Sequences (5′ → 3′)	T_ann_ [°C]	PrimerBank ID
*Actb*	F-Actb	GGCTGTATTCCCCTCCATCG	54	6671509a1
	R-Actb	CCAGTTGGTAACAATGCCATGT		
*HPRT*	F-HPRT	TCAGTCAACGGGGGACATAAA	54	7305155a1
	R-HPRT	GGGGCTGTACTGCTTAACCAG		
*SNORD115*	F-115	TCAATGATGACAACCCAATGTC	50	/
	R-115	CTCAGCGTAATCCTATTGAGCAT		
*SNORD116*	F-116	ATCTATGATGATTCCCAGTCAAACATTC	50	/
	R-116	GATGAGAGTGGCGGTACAG		
*SNORA35*	F-35	CAAGTGACCCATTGGACTTTTTCTTG	52	/
	R-35	TGTGACTCTCCCATCTTTCGG		
*SNORD45B*	F-45B	ATGGCATGTATTAGCTGAATGCTAACC	50	/
	R-45B	AAGGTCTCAGTGTAATTTGTAACTTGC		
*5ht2c* (long isoform)	F-5HT2c_long	GCCATCATGAAGATTGCCATCGTTTG	55	/
	R-5HT2c_long	CACGCAGGTAGTATTATTCACGAACACT		
*5ht2c* (short isoform)	F-5HT2c_short	ATCGCTGGACCGGAGTTTC	55	/
	R-5HT2c_short	GTTCGGGTCATTGAGCACGC		
*ADAR1*	F-Adar1	CAGAGGCACTGTGGATGGAC	54	/
	R-Adar1	CATAGTCACGGGCAGCTTTCTTG		
*ADAR2*	F-Adar2	GTGACAAGATAGCACGCTGGAAC	54	/
	R-Adar2	TATGTTGGAGATCCGCTGGTACATG		

### Analysis of alternative splicing for *5ht2c* transcripts

Potential changes in *5ht2c* alternative exon Vb usage during P19 neuronal differentiation were analysed by qPCR with primers given in Table 2. Additionally, ratio of long to short receptor isoform was assessed by touchdown end-point PCR. Here, 22 ng of cDNA was used per 15 µL reaction. Primers F1-m5HTR2c (5′-CTAGATATTTGTGCCCCGTCTG-3′) and R1-m5HT2cR (5′-GTCATTGAGCACGCAGGTAGTA-3′) were used at 200 nM concentration and PCR conditions were: 30 cycles of 30 s at 95 °C, 2 min at 60 °C with a decrease of 0.5 °C each cycle, and 2 min at 72 °C, followed by 15 cycles of 30 s at 95 °C, 1 min at 45 °C and 1 min at 72 °C, and a final extension step of 7 min at 72 °C. Amplicons were separated on 2.5% agarose gel (one third of the reaction was loaded) and stained with ethidium bromide. Stain fluorescence intensities were quantified on G Box imager (Syngene, Cambridge, UK) and analysed with GeneTools software, normalizing signals to amplicon length.

### Analysis of A-to-I editing

A-to-I editing levels were quantified by sequencing. The 280-nucleotide *5ht2c* gene segments encompassing editing sites A to D were amplified using primers F2-m5HT2cR (5′-CAATGCTACCAATTATTTCTTAATGTC-3′) and R2-m5HT2cR (5′-AACGATGGCAATCTTCATG-3′). 50 μL reactions were performed with 40 ng cDNA at 35 cycles of 20 s at 95 °C, 30 s at 51 °C, and 1 min at 72 °C. Amplicons were purified using QIAquick PCR purification Kit (Qiagen, Venlo, Netherlands) and sequenced using primer F2-m5HT2cR. The percentage of adenosine conversion to inosine at individual sites was calculated as $\% I=\,\frac{G}{G+A}\times 100$, where G is guanosine peak height and A is adenosine peak height.

A recent study indicated discrepancies in the extent of editing determined from peak height ratios (direct PCR sequencing) and distribution of NGS counts at high and low editing levels^34^. To check the validity of our data, we sequenced defined mixtures of two *5ht2c* clones, subcloned in pGem-T Easy plasmid (Promega, Madison, WI, USA), differing in A-to-I editing (i.e. A to G transitions) at 3 positions.

### Statistical analyses

For the qPCR expression analyses, data (relative gene expression normalized to geometric mean of *Actb* and *HPRT*) are presented as mean values for 3 biological repeats in each group ± standard deviation. For the quantitative splicing analyses of *5ht2c* transcript, percentage of long splice isoform in each sample was calculated (from qPCR or densitometric measurements) and averaged across each group of 3 biological repeats. Data are expressed as average values ± standard deviation. Groups were compared with one-way ANOVA followed by Bonferroni *post hoc* test (GraphPad Prism, GraphPad Software, CA, USA). For quantitative A-to-I editing analyses of *5ht2c* transcript, percentage of inosine at each of the five positions for each sample was calculated and averaged across each group of 3 biological repeats. Data are expressed as average values ± standard deviation. The extent of A-to-I editing at each of the five positions during neuronal differentiation was compared to that of undifferentiated P19 cells with Kruskal-Wallis one-way analysis of variance followed by Dunn’s *post hoc* test (GraphPad Prism).

## Electronic supplementary material

Supplementary Information
