# Supplementary material for: Neuronal differentiation induces SNORD115 expression and is accompanied by post-transcriptional changes of serotonin receptor 2c mRNA
Source: Sci Rep. 2018 Mar 23;8:5101. doi: 10.1038/s41598-018-23293-7 (PMC5865145; doi:10.1038/s41598-018-23293-7)
Supplement: Supplementary file 1 — Supplementary Information [file 41598_2018_23293_MOESM1_ESM.docx]

*Supplementary information*

**Neuronal differentiation induces SNORD115 expression and is accompanied by post-transcriptional changes of serotonin receptor 2c mRNA**

Tomaž Bratkovič^1^, Miha Modic^2^, Germán Camargo Ortega^2^, Micha Drukker^2^ & Boris Rogelj^3,4,5,*^

^1^ University of Ljubljana, Faculty of Pharmacy, Department of Pharmaceutical Biology, Aškerčeva 7, 1000 Ljubljana, Slovenia

^2^ Institute of Stem Cell Research, German Research Center for E nvironmental Health, Helmholtz Center Munich, 85764 Neuherberg, Germany

^3^ Jozef Stefan Institute, Department of Biotechnology, Jamova 39, 1000 Ljubljana, Slovenia

^4^ Biomedical Research Institute BRIS, Puhova 10, 1000 Ljubljana, Slovenia

^5^ University of Ljubljana, Faculty of Chemistry and Chemical Technology, Večna pot 113, 1000 Ljubljana, Slovenia

* Correspondence: Boris Rogelj ([boris.rogelj@ijs.si](mailto:boris.rogelj@ijs.si))

***Supplementary figures***

**Supplementary Figure S1.** Representative electropherograms of *5ht2c* Sanger sequencing for each time point during neuronal differentiation.

**Supplementary Figure S2.** Increase in *ADAR1* and *ADAR2* gene expression during neuronal differentiation as deduced by qPCR. Averaged data ± standard deviation are shown.

**Supplementary Figure S3.** Accuracy of extent of A-to-I editing determination by direct Sanger amplicon sequencing. Plasmids harboring two *5ht2c* clones differing in A to G transition at 3 sites were mixed at different ratios and sequenced to compare the actual and deduced levels of A to G transition. Averaged data ± standard deviation are shown. For G to A ratios in the range of 20:80 to 80:20, the curve is linear. At lower and higher ratios, respectively, the shape of the calibration curve turns sigmoidal, allowing somewhat less accurate determination of editing extent; consistent with findings of Wahlstedt et al. [^1^](#_ENREF_1).

**Supplementary Figure S4**. Example of qPCR amplification curves (for SNORD116). Amplification curves for 4 individual samples (smaller panels above) and the entire set of 12 samples (larger panel below) are shown. Each sample was analysed in 3 technical repeats. No reverse transcription controls gave rise to curves shifted to the right relative to cDNA containing samples of equal dilutions. Differences in Cp values between no reverse transcription controls and cognate reverse-transcribed samples were in the range of ~7 to ~16, suggesting genomic DNA contamination relative to RNA levels well below 1%. Green curves depict no template control.

**Supplementary Figure S1**

**
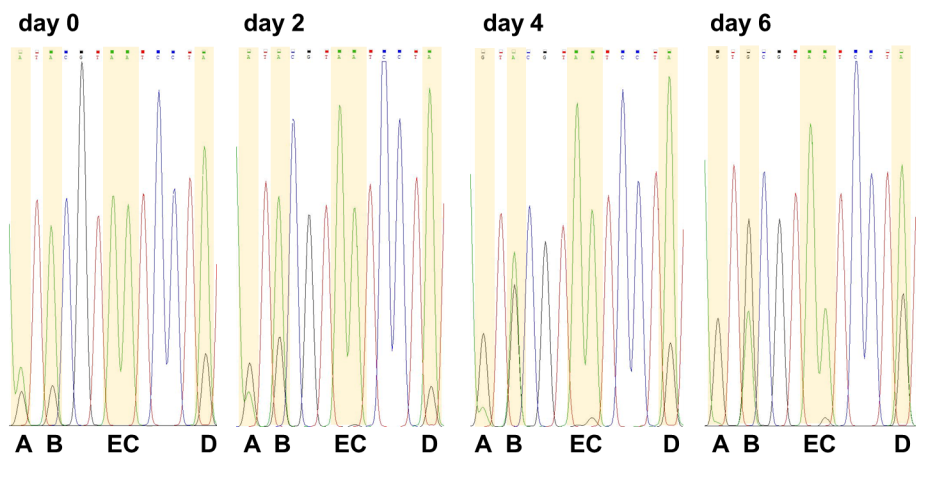
**

**Supplementary Figure S2**

**
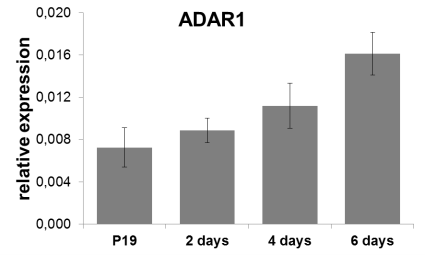

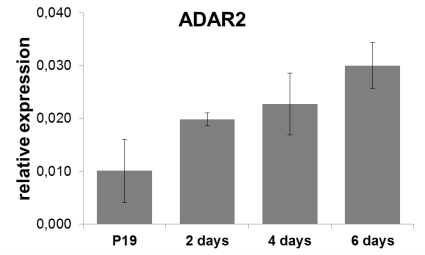
**

**Supplementary Figure S3**

**
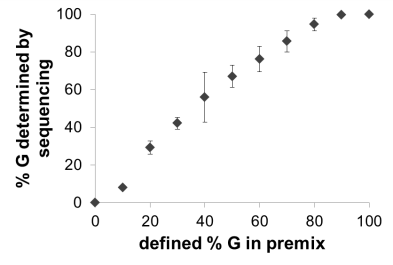
**

**Supplementary Figure S4**

**
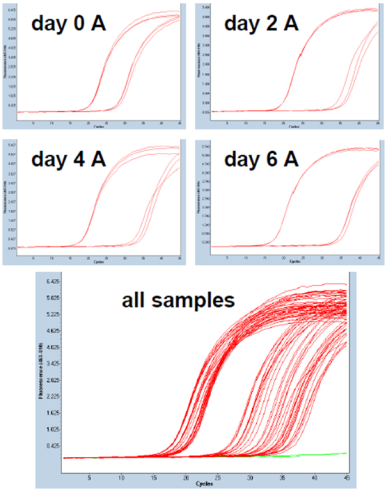
**

**Reference**

1. Wahlstedt, H., Daniel, C., Enstero, M. & Ohman, M. Large-scale mRNA sequencing determines global regulation of RNA editing during brain development. *Genome Res* 19, 978-986 (2009).
